# Immunolymphoscintigraphy for Metastatic Sentinel Nodes: Test of a Model

**DOI:** 10.1155/2011/828151

**Published:** 2011-04-28

**Authors:** A. H. Chakera, B. S. Nielsen, J. Madsen, J. Romer, P. E. G. Kristjansen, I. Buch, T. Binderup, C. Ingvar, A. Nalla, A. Kjaer, B. Hesse

**Affiliations:** ^1^Clinic of Plastic Surgery, Breast Surgery and Burns Unit, Rigshospitalet, Blegdamsvej 9, 2100 Copenhagen, Denmark; ^2^The Finsen Laboratory, Rigshospitalet, 2100 Copenhagen, Denmark; ^3^Diagnostic Product Development Division, Exiqon A/S, 2950 Vedbaek, Denmark; ^4^Clinic of Clinical Physiology, Nuclear Medicine, and PET, Rigshospitalet, 2950 Copenhagen, Denmark; ^5^Novo Nordisk A/S, 2880 Bagsvaerd, Denmark; ^6^Institute of Molecular Pathology, University of Copenhagen, 1165 Copenhagen, Denmark; ^7^Faculty of Medical Laboratory Science, Metropolitan University College, 1150 Copenhagen, Denmark; ^8^Cluster for Molecular Imaging, Faculty of Health Sciences, University of Copenhagen, 1165 Copenhagen, Denmark; ^9^Department of Surgery, Lunds University Hospital, 221 85 Lund, Sweden; ^10^Department of Biomedical Sciences, Faculty of Health Sciences, University of Copenhagen, 1165 Copenhagen, Denmark

## Abstract

*Aim*. To develop a method and obtain proof-of-principle for immunolymphoscintigraphy for identification of metastatic sentinel nodes. *Methods*. We selected one of four tumour-specific antibodies against human breast cancer and investigated (1), in immune-deficient (nude) mice with xenograft human breast cancer expressing the antigen if specific binding of the intratumorally injected, radioactively labelled, monoclonal antibody could be scintigraphically visualized, and (2) transportation to and retention in regional lymph nodes of the radioactively labelled antibody after subcutaneous injection in healthy rabbits. *Results and Conclusion*. Our paper suggests the theoretical possibility of a model of dual isotope immuno-lymphoscintigraphy for noninvasive, preoperative, malignant sentinel node imaging.

## 1. Introduction

The most significant prognostic factor in breast cancer, melanoma, and other solid tumours with primarily lymphatic spread is the metastatic status of the regional lymph nodes. Therefore, identification of possible subclinical regional lymph node metastases is of major importance. The sentinel node (SN) technique is now widely accepted as a minimally invasive and accurate method for identifying breast cancer and melanoma patients with subclinical regional lymph node metastases likely to benefit from complete regional lymph node dissection (CLND) and possibly adjuvant therapy. The technique saves approximately 60% of breast cancer patients [1] and 80% of melanoma patients [2, 3] without regional metastases from the risk of complications related to CLND. The SN is usually defined as any lymph node receiving direct lymph from the primary tumour site, and it is thought to be the entrance for further regional spread of the cancer, followed by delayed progression of metastases to other lymph nodes in the region [4]. 

The use of lymphoscintigraphy and subsequent intraoperative probe detection and use of blue dye for visual SN identification have improved patient management, morbidity, and probably also mortality. An important limitation of SN detection is the lack of knowledge before the operation as to whether the SN contains malignant cells or not. The retention in the regional lymph nodes of colloids used for SN lymphoscintigraphy only identifies the SN but is not related to the presence of malignant cells in the SNs. Excision of an SN, therefore, has to be combined with histology and immunohistochemistry to identify possible malignant cells. 

If malignant tissue in the regional lymph nodes could be detected preoperatively and noninvasively, permitting better selection of patients for CLND, the SN technique would be clinically greatly improved. This could lead to omission of regional lymph node surgery in node-negative patients and diminishing the extent of surgery with better preoperative planning of a one-stage procedure in node-positive patients. Immunolymphoscintigraphy might represent such a tool. Immunolymphoscintigraphy refers to scintigraphy with interstitial (subcutaneous, intracutaneous, or intratumoral) injection of a tumour-specific radiolabelled antibody for malignant lymph node imaging. A variety of radiolabelled antibodies against tumour-associated antigens have previously been studied, both in animals and humans, to test their ability to selectively target cancer cells in lymph nodes. In the immunoscintigraphy literature, more interest has been paid to intravenous administration of radiolabelled antibodies, often with disappointing results [5, 6], though a few promising studies have been reported [7, 8]. By intravenous administration of the radiolabelled antibody, only a small fraction of the antibody gets direct access to the target, partly due to the distribution of the antibody in the total blood volume, and partly due to a generally high and rapid hepatic uptake of foreign proteins. Therefore, a lymphoscintigraphy approach with interstitial injection of the antibody appears promising in several diseases for detection of metastatic regional lymph nodes [9, 10]. Results reported have been promising, but not yet sufficiently efficient for clinical use [11–13]. Will the antibody injected like a sentinel node radiopharmaceutical pass to the regional lymph nodes, but only stay there for a limited time interval permitting binding to the specific antigen if present on the surface of tumour cells?

The aim of the study was to develop a method and obtain proof-of-principle for immunolymphoscintigraphy for identification of metastatic sentinel nodes. To do so we (1) in vitro tested and selected one of four tumour specific antibodies against membrane bound antigens expressed by human breast cancer, (2) investigated the in vivo antigen-antibody binding in a xenograft mice model with human breast cancer, known to express the antigen and with tumours sized-like human lymph nodes, and (3) investigated if radioactively labelled antibodies were transported to and through regional lymph nodes when injected subcutaneously in healthy rabbits. A strategy to set up dual isotope immunolymphoscintigraphy for noninvasive, preoperative, malignant sentinel node diagnosis is finally suggested.

## 2. Materials and Methods

### 2.1. In Vitro Test of Antibodies on Tissue and Cell Lines

**Figure 1:**
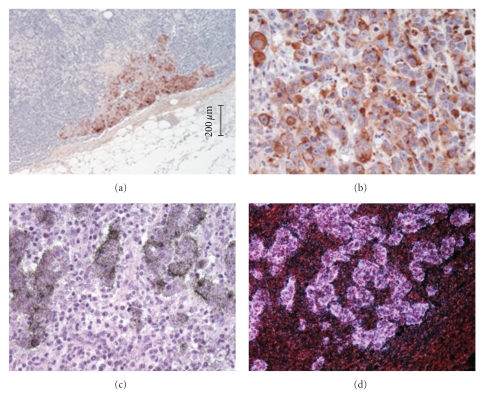
The figure shows specific immunostaining of MCF-7 tumour metastases in human lymph nodes. There is no staining of normal lymph node tissue; (a and b): immunoperoxidase staining with anti-MUC1 (2 different views) (c and d) ^125^I-immunohisto-autoradiogrphy with anti-MUC1, (c) bright-field, (d) dark field.

We tested 4 different murine monoclonal antibodies known to be expressed in human breast cancer; 3 against polymorphous epithelial mucin (MUC1), an epithelial transmembrane glycoprotein (clone E29 (Dako, Glostrup, Denmark), clone GP 1.4 (AbCam, Cambridge, UK), and clone 115D8 (Monosan, Uden, The Netherlands)), and one against epithelial cellular adhesion molecule (EpCam (Calbiochem, Merck, Darmstadt, Germany)). Binding of the 4 antibodies to human MCF-7 and MDA-MB-231 breast cancer cells and lymph nodes with and without metastases were tested by immunoperoxidase staining using chromogenic identification and radioactive labelling with iodide-125 (^125^I) followed by autoradiography (Figure 1). In brief, tissue sections were predigested with proteinase-K and incubated with the ^125^I-labelled antibody (see below) or nonlabelled antibody at 1–5 *μ*g/mL for one hour and subsequently washed several times with PBS. The tissue sections incubated with the radiolabelled antibodies were dried in ethanol and immersed into an autoradiographic emulsion (Ilford Imaging, Knutsford, UK) and exposed 1–3 days before development. The tissue sections with nonlabelled antibodies were immunoperoxidase stained using mouse-specific Envision reagent (Dako, Glostrup, Denmark) followed by NovaRed chromogen development (Vector Laboratories, Burlingname, CA, USA). Among the 4 monoclonal antibodies the anti-MUC1, Clone 115D8, (MON 9005, Monosan, Uden, The Netherlands) showed the highest signal-to-noise ratio in human breast tissue sections and stained virtually all MCF-7 cells, and, therefore, was chosen for in vivo studies.

### 2.2. Radiolabelling of Anti-MUC1

Anti-MUC1, Clone 115D8, was presented as purified IgG at approximately 100 *μ*g IgG/mL in azide-free PBS. Mouse monoclonal antibody against trinitrophenyl hapten (TNP) [14] diluted in azide-free PBS was kindly provided by Dr. Gunilla Hoyer-Hansen (The Finsen Laboratory, Rigshospitalet, Denmark). Iodide-125 (^125^I) labelling of anti-MUC1 was used for immunohistochemical analyses. Iodide-131 (^131^I) was chosen for the first series of in vivo studies due to its long half-life. Iodide-123 (^123^I) was used in the second series because of its better energy spectrum for imaging purposes, compared with ^131^I. Antibodies were radiolabelled with ^123^I,^ 125^I, and ^131^I using the iodogen method [15]. Free radioactive iodide was removed using a Dowex 1 × 8–100 anion exchange resin. According to thin-layer chromatography of the radiolabelled antibodies, radiochemical purity above 95% was obtained.

### 2.3. Anti-MUC1/-TNP (^123^I and ^131^I) In Vivo Studies in Mice

Two test series were performed in 6-week-old female NMRI nude mice (*n* = 21) inoculated with 10^7^ MCF-7 cells bilaterally in the thigh. One week prior to inoculation with MCF-7 cells, a 17*β*-estradiol pellet (0.72 mg, 60-day release, Innovative Research of America, Sarasota, FL, USA) was implanted subcutaneously in the neck. Upon inoculation, MCF-7 cells were suspended in an equal amount of Matrigel (BD Biosciences, Broendby, Denmark) to support growth of the tumours. When tumours were 0.5–1 cm^3^ in size, the radioactively labelled antibody was injected intratumorally; either anti-MUC1 or the nonspecific anti-TNP as control in a volume of approximately 50 *μ*L with insulin syringe. ^131^I was used in 9 mice and ^123^I in 12 mice. In the ^131^I series, each tumour was injected with median 3 MBq (range 1–18) ^131^I-labelled antibodies. In the ^123^I series, each tumour was injected with 10 MBq ^123^I-labelled antibodies. One (^123^I series) or 3 (^131^I series) days after injection, the mice were sacrificed, and the biodistribution of the radioactively labelled antibodies was studied on a gamma camera (ProVivo, Santax Medico, Glostrup, Denmark) with 30 minutes static images. For ^123^I, a low-energy, parallel-hole, general-purpose collimator was used, for ^131^I, a medium-energy, parallel-hole, general-purpose collimator was used. In the ^131^I series, the tumours, thyroid gland, kidneys, liver, and a foot (reference tissue) were dissected and weighed after 7 days, and final imaging and count rates (cps/g tissue) were determined in a well counter.

### 2.4. ^99m^Tc-HIG/-Nanocolloid In Vivo Studies in Rabbits

Four healthy female New Zealand white rabbits (weight 2.5–2.9 kg) were anaesthetized with Hypnorm-Dormicum subcutaneously. Two rabbits were injected subcutaneously on the front side of each lower leg with 40 MBq technetium-99 m-labelled human immunoglobulin G (^99m^Tc-HIG) (Mallinckrodt, Petten, The Netherlands) and 2 with 40 MBq technetium-99 m-labelled human albumin nanocolloid (^99m^Tc-nanocolloid Nanocoll, Tyco, Rotterdam, The Netherlands) as control. ^99m^Tc labelling of HIG was chosen in this pilot study because HIG consists of polyclonal IgG molecules with a size similar to anti-MUC1 and because a simple kit for ^99m^Tc labelling exists. Images of the rabbits were acquired on a 1-headed gamma camera (Mediso Medical Imaging Systems, Budapest, Hungary) with a low energy, general-purpose collimator 15 minutes and 1, 2.5, 5, and 22 hours after radioisotope injection (hours p.i.). Dynamic images (matrix size 256 × 256, 5 min. per region) were used during the first 2.5 hours p.i. with separate image acquisition over both injection sites and over the inguinal lymph nodes. Static images (matrix size 128 × 128, 10 min. per region) were used at 5 and 22 hours p.i. with image acquisition over both injection sites, inguinal lymph nodes and the abdomen. Regions of interest (ROIs) were drawn around the injection sites, the inguinal lymph nodes, the bladder, the kidneys, the liver, and the spinal column. Count rates were corrected for physical decay and background. Labelling yield of ^99m^Tc-HIG and ^99m^Tc-nanocolloid were checked with paper chromatography, and purity above 95% was obtained.

### 2.5. Statistics

This study was set up as a proof-of-concept examination with few animals included in each step. Generally, the statistical analysis, is therefore, absent or very limited and only nonparametric methods were used. 

## 3. Results

### 3.1. Cellular In Vitro Tests

Immunohistoautoradiography with ^125^I-anti-MUC1 (Clone 115D8) recognizing the epithelial-specific surface protein MUC1 showed the highest signal-to-noise ratio among 4 monoclonal antibodies tested and showed specific binding to MCF-7 cells and no binding to normal lymph node tissue (Figure 1).

### 3.2. Tumoral Antibody Retention in Mice

**Figure 2:**
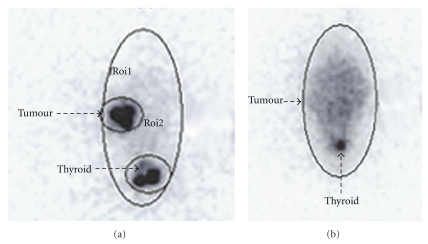
High tumour retention of ^131^I-anti-MUC1 in a mouse after intratumoral injection (a) and no tumour retention of  ^131^I-anti-TNP in a mouse after intratumoral injection (b).

Tumoral retention of ^131^I-anti-MUC1 was visualized (Figure 2) with gamma camera images 72 hours p.i. in 2 of 5 mice (one mouse died). No tumour retention of unspecific ^131^I-anti-TNP antibody was found in any of the 3 control mice. Count rates per weight tissue (cps/g) determined in the well counter and calculation of tumour/foot ratio (foot serving as reference region) showed ^131^I-anti-MUC1 retention in 4 of the 5 mice (median 14 cps/g, range 1–109) and low tumour retention of  ^131^I-anti-TNP (median 1 cps/g, range 1–6). Furthermore, uptake of both ^131^I-anti-MUC1 and ^131^I-anti-TNP was shown in the thyroid gland in all mice (Table 1). 

In the ^123^I series, gamma camera images showed uncertain tumour retention of ^123^I-anti-MUC1, comparable with ^123^I-anti-TNP retention. Diffuse uptake in abdominal organs and high thyroid ^123^I uptake, as an indirect sign of deiodination, were seen with both antibodies.

### 3.3. Lymphatic Drainage of Immunoglobulins and Colloids in Rabbits

**Figure 3:**
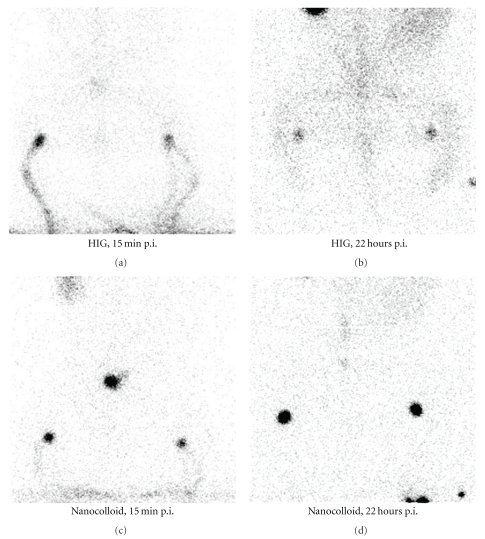
Visualization on gamma camera images of groin lymph nodes 15 min. and 22 hours after injection in rabbits of ^99m^Tc-HIG and ^99m^Tc-nanocolloid, respectively.

**Figure 4:**
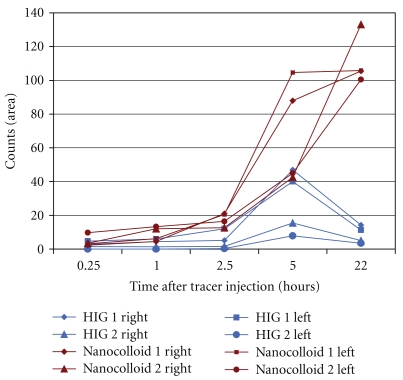
Uptake of  ^99m^Tc-nanocolloid and ^99m^Tc-HIG in the groin lymph nodes of 4 rabbits over time.

Transport of both ^99m^Tc-HIG and ^99m^Tc-nanocolloid from the subcutaneous depots towards the inguinal lymph nodes was observed in the gamma camera images (Figure 3). ^99m^Tc-nanocolloid accumulated over hours in the lymph nodes with maximum at 22 hours p.i. Gradually, but fairly consistently, some hepatic uptake was observed. ^99m^Tc-HIG passed more rapidly through the nodes with maximum at 5 hours p.i., and activity gradually increased in the liver and in the spine (Table 2 and Figure 4, liver and spine data not shown). 

## 4. Discussion

The two steps of our study strongly suggest that malignant SN imaging is possible. Using mice with human breast cancer xenografts and gamma camera imaging, we showed the possibility of noninvasive demonstration of increased retention of a radiolabelled, tumour-specific antibody compared with a nonspecific radiolabelled antibody. Intratumoral injection was used in the mouse studies to minimize impact of changed drainage patterns of xenograft tumours, and washout was evaluated by determining counts/g in a well counter of tumours and several organs. Gamma camera images of the rabbits after subcutaneous injection showed that radiolabelled, polyclonal IgG is drained by the lymphatic vessels, but in contrast to the larger colloid particles like the nanocolloids used for SN imaging, IgG is cleared over hours from regional nonmetastatic lymph nodes. This has also been shown by others [9, 11, 12]. Finally, our findings with ^123^I-labelled anti-MUC1 demonstrated the well-known observation that iodine-labelled antibodies may not be stable in vivo, in spite of excellent in vitro purity, due to in vivo deiodination [16]. The difference between the tumour retention and higher deiodination of ^123^I-compared to the ^131^I-labelled antibody is not obvious. It may be related to difference in specific radioactivities of the iodine-labeled products. 

The appropriate use of radiolabelled antibodies in the diagnostic evaluation of cancer patients has not yet been clearly defined. A large number of studies used intravenous injections [5–8], but several challenges have caused a low sensitivity of successful imaging of malignant tumours with intravenous administration of radiolabelled antibodies. Due to the radiation exposure to the body, especially related to high liver and bone marrow uptake, the radioactive amount possible to inject intravenously is limited, thereby limiting the possible fraction taken up by the tumour and hence target-to-background ratio. Another problem related to intravenous administration is the risk of a high hepatic extraction fraction of the antibody [17]. Therefore, the low count rate of the small fraction of the antibody reaching the target (lymph nodes) will be too low to visualize radioactive binding to malignant tissue in the lymph nodes. Finally, possible cross-reaction with the injected antibody against circulating antigens [17] might sometimes reduce the available amount of radiolabelled antibody for binding to possible malignant tissue in the lymph node. 

These problems can be largely circumvented using local interstitial (subcutaneous, intratumoral, or peritumoral) injection of the radiolabelled antibody by presenting smaller amounts of radiolabelled monoclonal antibody closer to the metastasis, thereby increasing target-to-background ratio. (Immunoglobulins are macromolecules and when injected interstitially, preferentially drained by lymphatic vessels rather than the venous system). They then travel by lymph vessels to the regional lymph nodes where they can interact with the cells residing within the node (tumour cells, lymphocytes, or macrophages). Transit through the lymph nodes is delayed simply because of their large molecular size [18]. In contrast, radiocolloid particles used for SN scintigraphy are trapped within the lymph nodes due to uptake by macrophages in the lymph nodes [18]. Besides immunolymphoscintigraphy, interstitial injection of radiolabelled antibodies also raises the theoretical possibility of radiotherapy of regional malignant lymph nodes. Both deep and superficial injection techniques have been shown to be reliable for SN visualization in breast cancer. However extra axillary SNs are mainly visualized by intra- or peritumoral injection, whereas more axillary SNs are visualized by subcutaneous techniques [19]. A recent study of lymphatic anatomy on cadavers supports deep injection techniques [20].

Several different isotopes have been used for immunolymphoscintigraphy, each with their advantages and disadvantages. Deiodination in vivo is a well-known problem [16], as also demonstrated in our ^123^I mouse series. ^111^In-labelling of immunoglobulins may be a better choice than iodine labelling since ^111^In-labelled proteins are generally found to be stable in vivo [21], and the half-life of 2.7 days permits late imaging. Furthermore, given the energy spectrum of ^111^In (171 + 245 keV) this isotope, rather than the PET isotopes, could be a good candidate for combined use with a ^99m^Tc-labelled SN colloid for dual isotope SN imaging of metastatic infiltration of human SNs after local subcutaneous injection of both tracers. With the SN identified by gamma camera imaging using ^99m^Tc-labelled colloids, the gamma camera image can be highly focused on the region and thus much more sensitive, including pinhole collimator and SPECT or, even better, SPECT-CT images. Given the results of our mouse and rabbit studies, it might be possible to create a dual isotope SPECT or SPECT-CT technique with simultaneous standard SN lymphoscintigraphy and immunolymphoscintigraphy, which in case of presence of tumour cells could show tumour-specific antibody binding in SNs. If such a technique could be established, it would be a great improvement of the selection of patients for further surgery, avoiding SN biopsy in node-negative patients and directing node-positive patients directly to complete lymph node dissection. Of course, several issues still remain to be solved, including a high negative predictive value of the immunolymphoscintigraphy. The applicability of the technique will also depend on the minimum size of metastatic tissue that can be detected. The lower detection limit of immune-lymphoscintigraphy cannot be known in general since it will depend on a number of factors including the amount of antigen present on the surface of the relevant cancer cells, the binding affinity to the specific antigen, and the possible unspecific binding to other antigens present in the surrounding tissue, which in turn will determine the critical signal-to-noise ratio.

The small risk of developing human antimouse antibodies (HAMA) will probably only be relevant in patients receiving repeated injections of a murine monoclonal antibody, which could cause allergic responses and thus primarily interference with subsequent imaging or results of standard in vitro laboratory tests, and theoretically also a risk of anaphylactic complications [22]. Humanized monoclonal antibodies or small antibody fragments do not elicit a significant human antihuman (HAHA) response and should, therefore, be preferred for clinical use [22], but too small molecules may be less suited regarding lymphatic drainage. 

In addition to the limitation of having performed experiments only in very few animals, our proof-of-concept is also limited by the fact that we were not able to test the concept directly in one animal model, that is, a larger animal with a malignant tumour and metastases to the regional lymph nodes. Unfortunately, such an animal model was not available. However, we believed that with this initial proof-of-concept, a stepwise development could be achieved.

## 5. Conclusion

^131^I-labelled anti-MUC1 was found by gamma camera imaging to be retained with high specificity in xenograft human breast cancer tumours sized like a human lymph node, after intratumoral injection. Subcutaneous injection in rabbits of ^99m^Tc-labelled IgG showed drainage by the lymphatic vessels, but in contrast to colloids like the nanocolloids used for SN lymphoscintigraphy, they passed through regional, nonmetastatic lymph nodes with only limited delay. Iodine labelled antibodies may be less stable in vivo because of deiodination. An ^111^In-labelled tumour antibody might be a better candidate for a dual isotope technique for more specific, noninvasive, preoperative SN diagnosis in combination with standard ^99m^Tc-SN lymphoscintigraphy.

## Figures and Tables

**Table 1:** Count rates per second per gram tissue (cps/g) in different tissues from mice in the well counter after injection of  ^131^I-anti-MUC1 or ^131^I-anti-TNP. The foot is used as a reference region.

Antibody	Mouse no.	Tumour (cps/g)	Foot (cps/g)	Thyroid (cps/g)	Tumour/foot ratio
^131^I-anti-MUC1	1	1081	1627	1374	1
	2	7050	364	177	19
	3	171092	1563	2475	109
	4	90395	6546	7401	14
	5	22001	2472	2736	89
	Median				19

^131^I-anti-TNP	6	32284	23044	28172	1
	7	19526	10932	18764	2
	8	14519	2247	2376	6
	Median				2

**Table 2:** Mean percentages of activity in rabbit inguinal lymph nodes and liver as a ratio of activity at injection site at different times after injection, calculated from ROIs of the gamma camera images.

Percentage of injected activity (right + left foot)		Injection site	Inguinal lymph nodes	Liver
^99m^Tc-HIG: mean values from 4 rabbit extremities	15 min p.i.	100%	0 %	0%
	5 hours p.i.	53%	6%	4%
	22 hours p.i.	5%	2%	1%

^99m^Tc-nanocolloid: mean values from 4 rabbit extremities	15 min p.i.	100%	1%	0%
	5 hours p.i.	77%	9%	4%
	22 hours p.i.	42%	14%	3%
